# Supplementary material for: Ecomorphospace occupation of large herbivorous dinosaurs from Late Jurassic through to Late Cretaceous time in North America
Source: PeerJ. 2022 Apr 11;10:e13174. doi: 10.7717/peerj.13174 (PMC9009330; doi:10.7717/peerj.13174)
Supplement: File S1 [file peerj-10-13174-s001.pdf]

Complete list of institutional abbreviations

AMNH, American Museum of Natural History, New York, New York; CEUM, College of Eastern Utah Prehistoric Museum, Price, Utah; CM, Carnegie Museum of Natural History, Pittsburgh, Pennsylvania; CCM, Carter County Museum, Ekalaka, Montana; CMN, Canadian Museum of Nature, Ottawa, Ontario; DINO, DNM, Dinosaur National Monument, Jensen, Utah; DMNH, Denver Museum of Nature and Science, Denver, Colorado; FMNH, Field Museum of Natural History, Chicago, Illinois; FWMSH, Fort Worth Museum of Science and History, Fort Worth, Texas; GMNH-PV, Gunma Museum of Natural History Vertebrate Paleontology, Gunma, Japan; GPDM, Great Plains Dinosaur Museum, Malta, Montana; HMNS, Hayashibara Museum of Natural Sciences, Okayama, Japan; LL, cast housed in the University of Manchester Museum; MOR, Museum of the Rockies, Bozeman, Montana; MSC, McWane Science Center, Birmingham, Alabama; NCSM, North Carolina Museum of Natural Sciences, Raleigh, North Carolina; NHMUK, Natural History Museum, London, U.K.; NMMNH, New Mexico Museum of Natural History, Albuquerque, New Mexico; NSM PV, National Museum of Nature and Science, Tokyo, Japan; OMNH, Sam Noble Oklahoma Museum of Natural History, University of Oklahoma, Norman, Oklahoma; OTM, Old Trail Museum, Choteau, Montana; RAM, Raymond M. Alf Museum of Paleontology, Claremont, California; ROM, Royal Ontario Museum, Toronto, Ontario; SM, Senckenberg Museum, Frankfurt, Germany; SMA, Sauriermuseum Aathal, Aathal, Switzerland; SMU, Shuler Museum of Paleontology, Southern Methodist University, Dallas, Texas; TATE, Tate Geological Museum, Casper College, Casper, Wyoming; TMM, Texas Memorial Museum, Austin, Texas; TMP, Royal Tyrrell Museum of Palaeontology, Drumheller, Alberta; UALVP, University of Alberta, Edmonton, Alberta; UCMP, University of California Museum of Paleontology, Berkeley, California; UKMNH,

University of Kansas, Museum of Natural History, Lawrence, Kansas; UMMP, University of Michigan Museum of Paleontology, Ann Arbor, Michigan; USNM, Smithsonian Institution, National Museum of Natural History, Washington, D.C.; VFSMA, Verein für das Sauriermuseum, Aathal, Switzerland; YPM, Peabody Museum of Natural History, Yale University, New Haven, Connecticut; YPM PU, Peabody Museum of Natural History, Yale University, New Haven, Connecticut, Princeton University Collection.

Software used

Harrington B et al. 2004. Inkscape. Available at http://www.inkscape.org/ (accessed 22 November 2021).

RStudio Team. 2015. RStudio: Integrated Development for R. RStudio, Inc., Boston, MA. Available at http://www.rstudio.com/ (accessed 22 November 2021).

Schneider CA Rasband WS, Eliceiri KW. 2012. NIH Image to ImageJ: 25 years of image analysis. *Nature methods* 9(7): 671-675. PMID 22930834.

R packages used

Packages used to import datasets from excel and manipulate data frames

Bryan J. 2019. readxl: Read Excel Files. R package version 1.3.1. Available at https://CRAN.R-project.org/package=readxl (accessed 22 November 2021).

Dragulescu A and Arendt C. 2020. xlsx: Read, Write, Format Excel 2007 and Excel 97/2000/XP/2003 Files. R package version 0.6.5. Available at https://CRAN.R-project.org/package=xlsx (accessed 22 November 2021).

Henry L, Wickham H, RStudio. 2020. purrr: Functional Programming Tools. R package version 0.3.4. Available at https://CRAN.R-project.org/package=purrr (accessed 22 November 2021).

Wickham H. 2011. plyr: Tools for Splitting, Applying and Combining Data. R package version 1.8.6. Available at https://CRAN.R-project.org/package=plyr (accessed 22 November 2021).

Wickham H. 2020. reshape2: Flexibly Reshape Data: A Reboot of the Reshape Package. R package version 1.4.4. Available at https://CRAN.R-project.org/package=reshape2 (accessed 22 November 2021).

Wickham H, François R, Henry L, Müller K. 2018. dplyr: A Grammar of Data Manipulation. R package version 0.7.6. Available at https://CRAN.R-project.org/package=dplyr (accessed 22 November 2021).

Wickham H and RStudio. 2019. tidyverse: Easily Install and Load the 'Tidyverse'. R package version 1.3.0. AVAILABLE AT https://CRAN.R-project.org/package=tidyverse (accessed 22 November 2021).

Packages used for constructing, combining and exporting plots

Bivand R, Rowlingson B, Diggle P, Petris G, Elgen S. 2021. splancs: Spatial and Space-Time Point Pattern Analysis. R package version 2.01-42. AVAILABLE AT https://CRAN.R-project.org/package=splancs (accessed 22 November 2021).

Edwards SM, Auguie B, Jackman S, Wickham H, Chang W. 2020. lemon: Freshing Up your ‘ggplot2’ Plots. R package version 0.4.5. AVAILABLE AT https://CRAN.R-project.org/package=lemon (accessed 22 November 2021).

Ihaka R, Murrell P, Hornik K, Fisher JC, Stauffer R, Wilke CO, McWhite CD, Zeileis A. 2021. colorspace: A Toolbox for Manipulating and Assessing Colors and Palettes. R package version 2.0-2. AVAILABLE AT https://CRAN.R-project.org/package=colorspace (accessed 22 November 2021).

Kassambara, A. 2020. ggpubr: ‘ggplot2’ Based Publication Ready Plots. R package version 0.4.0. AVAILABLE AT https://CRAN.R-project.org/package=ggpubr (accessed 22 November 2021).

Wickham H. 2016. ggplot2: Elegant Graphics for Data Analysis. R package version 3.3.0. AVAILABLE AT https://CRAN.R-project.org/package=ggplot2 (accessed 22 November 2021).

Analysis-specific packages

Used to conduct Bayesian PCA imputation for missing data and standard PCA

Stacklies W, Redestig H, Scholz M, Walther D, Selbig J. 2007. pcaMethods – a Bioconductor package providing PCA methods for incomplete data. *Bioinformatics* 23: 1164-1167. R package version 1.80.0.

Used to run omnibus and pairwise NPMANOVA.

Oksanen J, Guillaume Blanchet F. Friendly M, Kindt R, Legendre P, Dan McGlinn D, Peter R, Minchin PR, O'Hara RB, Simpson GL, Solymos P, Stevens MHH, Szoecs E, Wagner H. 2019. vegan: Community Ecology Package. R package version 2.5-6. Available from https://CRAN.R-project.org/package=vegan (accessed 22 November 2021).

Used to compute the harmonic mean p-value.

Wilson DJ. 2019. harmonicmeanp: Harmonic Mean p-Values and Model Averaging by Mean Maximum Likelihood. R package version 3.0. Available from ttps://CRAN.R-project.org/package=harmonicmeanp (accessed 22 November 2021).
